# Diagnosis and Treatment of Fungal Infections in Lung Transplant Recipients

**DOI:** 10.3390/pathogens12050694

**Published:** 2023-05-10

**Authors:** Jesus E. Escamilla, Spenser E. January, Rodrigo Vazquez Guillamet

**Affiliations:** 1 Department of Pharmacy, Barnes-Jewish Hospital, Saint Louis, MO 63110, USA; 2 Division of Pulmonary and Critical Care Medicine, Department of Medicine, Washington University School of Medicine, Saint Louis, MO 63110, USA; 3 Rodrigo Vazquez Guillamet, 4921 Parkview Place, Saint Louis, MO 63110, USA

**Keywords:** lung transplant, fungal infections, antifungals

## Abstract

Fungal infections are a significant source of morbidity in the lung transplant population via direct allograft damage and predisposing patients to the development of chronic lung allograft dysfunction. Prompt diagnosis and treatment are imperative to limit allograft damage. This review article discusses incidence, risk factors, and symptoms with a specific focus on diagnostic and treatment strategies in the lung transplant population for fungal infections caused by *Aspergillus*, *Candida*, *Coccidioides*, *Histoplasma*, *Blastomyces*, *Scedosporium/Lomentospora*, *Fusarium*, and *Pneumocystis jirovecii*. Evidence for the use of newer triazole and inhaled antifungals to treat isolated pulmonary fungal infections in lung transplant recipients is also discussed.

## 1. Introduction

Fungal infections are common in lung transplant recipients (LTR). The cumulative 1-year incidence of fungal infections in LTR ranges from 10–22% [1]. In a population-based cohort study of about 9200 solid organ transplant (SOT) recipients, LTR had the highest incidence of invasive fungal infections (IFI) at 43 per 1000 person-years and a 10-year probability of 26.4% [2]. In the setting of prophylaxis, the prevalence of IFI within 180 days of lung transplant is 19.1 per 100 surgeries, with *Aspergillus* spp. accounting for 58% of non-Candida IFI [3]. The elevated incidence of fungal infections in LTR is of significant consequence since it has been directly linked to the development of chronic lung allograft dysfunction (CLAD), which has been associated with poor 3- and 5-year outcomes in LTR [4]. Furthermore, IFI in LTR is associated with the highest 1-year mortality out of all SOT recipients [2]. Indeed, the identification and treatment of fungal infections in LTR are crucial to limit the poor long-term outcomes associated with this common post-transplant complication.

LTR are more prone to fungal infections compared to other solid organ transplant recipients due to increased exposure to microorganisms via direct contact of the allograft with the environment [5]. LTR allografts will also have diminished elimination of microorganisms through impairment in mucociliary clearance and cough reflex, and respiratory tract structural abnormalities secondary to chronic respiratory disorders predispose LTR to microorganism colonization [5,6]. Receipt of immunosuppression and a reduction of blood flow to the site of infection through airway ischemia also reduce the host immune system’s ability to defend against infections [5,7]. Diagnosis of fungal infections following lung transplantation poses unique challenges as it can be hard to distinguish between colonization and active infection [6]. The multifactorial nature behind the increased risk of fungal infections in LTR results in the enlistment of various strategies to prevent and manage active fungal infections. Antifungal prophylaxis, either universal or pre-emptive, combined with routine post-transplant surveillance, has been implemented as a strategy to mitigate the risk of fungal infections [7,8]. Additionally, once infected, the degree of immunosuppression may be lowered in combination with the administration of antifungal agents. Indeed, the identification and treatment of fungal infections in LTR are crucial to limit the poor long-term outcomes associated with this common post-transplant complication.

## 2. *Aspergillus*

*Aspergillus* is among one of the most common sources of fungal infections in LTR. The incidence of invasive pulmonary aspergillosis in LTR has been reported as occurring in approximately 4–8% of LTR and the utilization of antimold prophylaxis has extended the average time of onset to 550 days post transplant [9,10]. Patients are at an increased risk for infections from *Aspergillus* if they are colonized within the first 12 months post-lung transplantation, have a single lung transplant, ≥3 episodes of supratherapeutic tacrolimus levels, if they experience anastomotic complications, airway/graft ischemia, reperfusion injury, bronchial anastomotic leaks, airway narrowing, CMV infection, or have had episodes of allograft rejection [11,12]. Tracheobronchial disease is the most common manifestation, and dissemination to other organs may occur late after transplant.

### 2.1. Diagnosis

A recent consensus document on IA in SOT recipients states that the approach to diagnosis should be multifaceted including histopathology, microbiology, serology, and imaging but that definitive diagnosis is hampered by a lack of prospective and high-quality studies in SOT [12].

(1,3)-β-D-glucan is a component of the fungal cell walls of *Aspergillus*, *Candida*, and *Pneumocystis* species. The Fungitell^®^ assay is approved to detect (1,3)-β-D-glucan in the serum. A meta-analysis in mainly non-SOT immunocompromised patients demonstrated a sensitivity of 77% and specificity of 83% for IA [13]. In the lung transplant population, serum (1,3)-β-D-glucan was evaluated; there was no difference in median (1,3)-β-D-glucan values between those with and without IFI’s with a cutoff value of ≥60 pg/mL demonstrating a sensitivity of 64% with a specificity of 8%. False positives were linked to colonization of the respiratory tract and the receipt of renal replacement therapy within seven days of sample collection; false negatives were linked to the receipt of systemic antifungal therapy [14]. Two studies have evaluated (1,3)-β-D-glucan assays of the bronchoalveolar lavage (BAL) fluid in transplant recipients; median (1,3)-β-D-glucan values were similar between colonized and actively infected individuals [15]. One study utilized a cutoff of 41 pg/mL and found a sensitivity of 80% with 53% specificity [15]; the other study used a cutoff of 60 pg/mL with an associated sensitivity of 79% and specificity of 40% [16]. 

Galactomannan is a cell wall component of *Aspergillus* spp. that is released through the growth of the organism. Platelia™ *Aspergillus* antigen immunoenzymatic assay (EIA) can be used on serum or BAL fluid with a positive result if the serum or BAL fluid has an optical density (OD) of ≥0.5 [17]. Platelia™ assays have been demonstrated to have lower accuracy in SOT patients when compared to hematological malignancies or post-hematopoietic cell transplantation [17]. Specifically in LTR, using a positive OD cutoff of 0.5, 25% of patients with IA had a positive galactomannan serum sample; sensitivity marginally improved to 30% when the cutoff was raised to an OD of 0.66. Notably, most false positives occurred within the first two weeks following lung transplantation and sensitivity was 0% for detection of *Aspergillus* tracheobronchitis [18]. Galactomannan assays on BAL fluid to diagnose IA has been assessed in meta-analyses that mainly included non-lung transplant immunocompromised patients, demonstrating that it could be successfully employed with a positive OD cutoff value of 1.0 showing higher sensitivity and lower specificity than serum galactomannan assays [19]. In LTR, three studies have utilized a positive OD cutoff value of ≥0.5 in BAL fluid for IA diagnosis, demonstrating a sensitivity of 60–100% and specificity of 89–100% [20,21,22]. Another evaluation of the *Aspergillus* galactomannan assay on BAL fluid in LTR identified an optimal OD cutoff of 1.5 which gave a sensitivity of 100% and specificity of 90%; higher BAL galactomannan OD indexes were observed in single lung transplants when compared to bilateral transplants. With respect to false galactomannan results, receipt of piperacillin/tazobactam has historically been linked to false positive galactomannan results; however, recent publications have failed to show a significant association between false positive galactomannan results and receipt of piperacillin/tazobactam when multiple lots from varying manufacturers were assessed [23,24,25]. Cross-reactivity resulting in positive Platelia™ *Aspergillus* galactomannan results has been reported to occur in 50% of patients with *Paracoccidioides brasiliensis*, 67% of patients with *Histoplasma capsulatum*, 63% of those with *Cryptococcus neoformans*, and 37% of patients with *Cryptococcus gattii* infections [26].

PCR testing on BAL fluid in immunocompromised patients has been evaluated in a meta-analysis for diagnosing proven or probable IA and had a sensitivity of 75% with 94% specificity in the setting of heterogenous PCR techniques [27]. *Aspergillus* PCR testing has historically been limited by the inability to discriminate between colonization of the respiratory tract and active infection, the inability to determine *Aspergillus* subspecies, and the lack of a standardized PCR methodology. One study in LTR evaluated the use of real-time Viracor pan-*Aspergillus* PCR of BAL samples and compared results to BAL galactomannan assays in 137 patients. The optimal quantification cycle (Cq) for *Aspergillus* was determined to be ≤35, which led to a sensitivity of 100% with a specificity of 88% and was significantly lower than patients who were colonized with *Aspergillus*, although 81% of false positive PCR results were determined to be due to airway colonization and incidences of false positive PCRs were significantly higher than false positive galactomannan assay results [20].

A lateral flow device (LFD) qualitatively detects an *Aspergillus* extracellular glycoprotein antigen via a monoclonal antibody. In immunocompromised patients, BAL fluid was evaluated using LFD, galactomannan assay, PCR, (1,3)-β-D-glucan assay, and mycology culture; LFD demonstrated a sensitivity of 80% with 95% specificity [28]. In LTR, the use of LFD on BAL fluid showed similar results with a sensitivity of 91% and specificity of 83% for the detection of invasive pulmonary *Aspergillus* (IPA) [29]. A more recent study of BAL fluid point-of-care LFD in a heterogeneous immunocompromised population, including those critically ill, demonstrated a lower sensitivity (58–69%) and specificity (68–75%) for the diagnosis of IPA [30].

CT imaging of *Aspergillus* pulmonary infections within LTR has been studied with high-resolution computed tomography, which demonstrated that abnormalities were bilateral in 87%; among those with unilateral abnormalities, all were single lung transplants with the transplanted organ being affected in two out of three cases. Centrilobar tree-in-bud nodules with bronchial wall thickening were present in 65% of cases, consolidation and ground-glass opacities in 22%, and large nodules with or without halo sign were present in 13% of patients [31].

### 2.2. Treatment

Per IDSA guidelines, voriconazole is the first-line agent for the management of IA [32]. In a randomized controlled trial of 277 immunocompromised patients (mainly non-SOT) with definite or probable IA that compared voriconazole vs. amphotericin B, survival in the voriconazole group was 70.8% vs. 57.9% in the amphotericin B group (HR, 0.59, 95% CI, 0.40–0.88) [33]. The voriconazole group experienced fewer adverse drug reactions compared to the amphotericin B group, but also had an increased rate of transient visual disturbances. Isavuconazole is a second-generation triazole antifungal that has less drug–drug interactions with CYP3A4 substrates, such as tacrolimus and cyclosporine compared to other triazole antifungals, making it a desirable option for IA treatment in SOT recipients. However, there is limited evidence for its use following lung transplant. In the SECURE trial, which compared isavuconazole to voriconazole for the management of invasive mold infections, isavuconazole was shown to be non-inferior to voriconazole while also having a reduced incidence of hepatobiliary disorders, visual disorders, and skin or subcutaneous tissue disorders [34]. Twenty percent of patients were immunocompromised, but there were no lung transplant recipients. A non-comparative study in SOT described the use of isavuconazole for various mold infections, including 43 *Aspergillus* spp. infections [35]. Similarly, posaconazole has been identified as a therapeutic option for IA. A phase 3, randomized controlled trial identified that posaconazole was non-inferior to voriconazole in survival up to day 42, while also resulting in fewer treatment-emergent adverse events than voriconazole [36]. While there were no lung transplant recipients in the trial, there was a significant population of hematopoietic stem cell transplant recipients, and patients receiving treatment with T-cell immunosuppressants or prolonged courses of corticosteroids. Ultimately, while only voriconazole has been specifically studied in LTR, posaconazole or isavuconazole may be viable first-line alternatives given their more favorable safety profile, as well as a diminished effect of drug interactions with isavuconazole.

Alternative therapies may be considered in patients who are unable to tolerate voriconazole, posaconazole, or isavuconazole. Possible strategies include itraconazole; echinocandins such as caspofungin or micafungin; or lipid formulations of amphotericin B [32]. Table 1 contains information on antifungal interactions, adverse reactions, and recommended dosing for aspergillosis.

**Table 1:** Antifungal considerations in SOT.

Drug	Uses	Immunosuppressant Drug Interactions	Key Adverse Drug Reactions	Dosing (Pulmonary Infections)
Fluconazole	*Candida* (non-*glabrata*); *Cryptococcus*; *Coccidioides*; *Blastomyces* (alternative).	Tacrolimus: 50% increase in serum tacrolimus levels [37]. Sirolimus: 28–70% increase in serum sirolimus levels [38]. Everolimus: 2.8-fold decrease in everolimus clearance [39]. Cyclosporine: approximately 150% increase in serum cyclosporine levels [37].	QTc prolongation, hepatotoxicity [37]	*Candida* ^a^ [40]: 800 mg on day 1, followed by 400 mg daily; duration dictated by extent of dissemination and resolution of signs/symptoms. *Cryptococcus* ^b^ [41]: 400 mg daily for 6–12 months followed by chronic suppression. *Coccidioides* [42]: 400–1200 mg daily for 6–12 months followed by chronic suppression. *Blastomyces* ^b^ [43]: 400–800 mg daily for 6–12 months.
Itraconazole	*Aspergillus* (alternative); *Coccidioides*; *Histoplasma*; *Blastomyces*.	Tacrolimus: significant increases in concentrations requiring a 50–75% dose reduction [44]. Sirolimus: significant increase in sirolimus concentrations anticipated [45]. Everolimus: 3.9-fold increase in everolimus Cmax and 15-fold increase in everolimus AUC [46]. Cyclosporine: 50–75% cyclosporine dose reductions have been required in LTRs [47,48].	Hepatotoxicity, peripheral neuropathy, hearing loss, CNS depression, QTc prolongation [45]. **Boxed warning:** heart failure exacerbation through negative inotropic effects [45].	*Candida* ^c^ [45] Aspergillus^c,d^ [45]: 200–400 mg twice daily for 6–12 weeks. *Coccidioides* ^c^ [49]: 200 mg twice daily for ≥12 months followed by chronic suppression. *Histoplasma* ^c^ [42]: 200 mg twice daily for ≥12 months followed by chronic suppression. *Blastomyces* ^c^ [43]: 200 mg twice daily for 6–12 months.
Voriconazole	*Aspergillus*; *C. glabrata* (alternative); *C. krusei* (alternative); *Cryptococcus* (alternative); *Coccidioides* (alternative); *Histoplasma* (step-down, alternative) [50]. *Blastomyces* (alternative); *Scedosporium* (alternative); *Fusarium* [51].	Tacrolimus: 2-and 3-fold increases of tacrolimus Cmax and AUC, respectively [52]. Sirolimus: 4.5-to 11-fold increase in sirolimus AUC [52]. Everolimus: 8.2-fold increase in everolimus concentration/dose ratio; everolimus dose reductions of 67% have been needed [53,54]. Cyclosporine: 1.7-fold increase in cyclosporine AUC and 2.5-fold increase in cyclosporine minimum plasma concentration [52,55].	Acute kidney injury, QTc prolongation, hepatotoxicity, periosteal disease, and visual disturbances [52].	*Aspergillus* ^e,f^ [12], *Cryptococcus* ^f^ [56]: IV: 6 mg/kg twice daily for 2 doses, then 4 mg/kg twice daily; oral: 200 mg twice daily for ≥6 weeks. *Candida* ^f^ [40]: 400 mg twice daily for 2 doses, then 200–300 mg twice daily; duration dictated by extent of dissemination and resolution of signs/symptoms. *Coccidioides* ^f^ [57], *Histoplasma* ^f^ [52]: 400 mg twice daily for 2 doses, then 200 mg twice daily for ≥12 months followed by chronic suppression. *Blastomyces* ^f^ [4]: 400 mg twice daily for 2 doses, then 200 mg twice daily for 6–12 months. *Scedosporium* ^f^ [51]: IV: 6 mg/kg twice daily for 2 doses, then 4 mg/kg twice daily; oral: 400 mg twice daily for 2 doses, then 200–300 mg twice daily for a prolonged duration. *Fusarium* ^f,g^ [51]: IV: 6 mg/kg twice daily for 2 doses, then 4 mg/kg twice daily followed by step-down to oral 200 mg twice daily once improvement on IV for a prolonged duration.
Posaconazole	*Aspergillus* *Candida* (alternative); *Cryptococcus* (alternative); *Mucorales* (alternative); *Coccidioides* (alternative); *Histoplasma* (step-down, alternative); *Blastomyces* (alternative); *Fusarium* (alternative) [58].	Tacrolimus: ~120% increase in tacrolimus Cmax and ~350% increase in tacrolimus AUC [59,60]. Sirolimus: 8.9-fold increase in sirolimus AUC [61,62]. Everolimus: 3.5-fold increase in everolimus Cmin/dose ratio [63]. Cyclosporine: reductions in cyclosporine dose of 14–29% have been required [59].	Hepatotoxicity, QTc prolongation [64].	*Aspergillus* ^h^ [32]: tablets (preferred): 300 mg twice daily for 2 doses, then 300 mg daily; suspension: 200 mg three times daily or 400 mg twice daily for ≥6 months. *Candida* [65]: Tablet: 300 mg daily; suspension: 400 mg twice daily; duration dictated by extent of dissemination and resolution of signs/symptoms. *Cryptococcus* [57]: 300 mg twice daily for 2 doses, then 300 mg daily; suspension: 200 mg three times daily or 400 mg twice daily for 6–12 months followed by chronic suppression. *Mucorales* step-down [66], *Fusarium*: tablets/IV: 300 mg twice daily for 2 doses, then 300 mg daily (suspension not recommended) for a prolonged duration. *Coccidioides* [57], *Histoplasma* [67]: tablets: 300 mg twice daily for 2 doses, then 300 mg daily; suspension: 200 mg three times daily or 400 mg twice daily for ≥12 months followed by chronic suppression. *Blastomyces* [68]: tablets: 300 mg twice daily for 2 doses, then 300 mg daily; suspension: 200 mg three times daily or 400 mg twice daily for 6–12 months.
Isavuconazole	*Aspergillus* [35] *Candida* (alternative) [35]; *Cryptococcus* (alternative); *Mucorales* [35] (alternative); *Coccidioides* (alternative); *Histoplasma* (step-down, alternative); *Blastomyces* (alternative).	Tacrolimus: dose/concentration ratio has been decreased by 30% [69]. Sirolimus: likely to significantly increase sirolimus levels [70]. Everolimus: likely to significantly increase everolimus levels [71]. Cyclosporine: AUC and Cmax have been increased by 29% and 6%, respectively [62,71].	QTc shortening, hepatotoxicity [70].	*Aspergillus* [12]: 372 mg every 8 h for 6 doses, then 372 mg daily for ≥6 weeks. *Candida* [70]: 372 mg every 8 h for 6 doses, then 372 mg daily; duration dictated by extent of dissemination and resolution of signs/symptoms. *Cryptococcus* [72]: 372 mg every 8 h for 6 doses, then 372 mg daily for 6–12 months followed by chronic suppression. *Mucorales*: 372 mg every 8 h for 6 doses, then 372 mg daily for a prolonged duration [66]. *Coccidioides* [72], *Histoplasma:* 372 mg every 8 h for 6 doses, then 372 mg daily for ≥12 months followed by chronic suppression. *Blastomyces* [72]: 372 mg every 8 h for 6 doses, then 372 mg daily for 6–12 months.
Caspofungin	*Aspergillus* (alternative) [32]. *Candida* *Mucorales* (alternative in combination with amphotericin B) [66].	Tacrolimus decrease in Cmax by 16%, Cmin by 26%, and AUC by 20% [73].	Hypotension, peripheral edema, tachycardia, phlebitis, and elevated liver enzymes [73].	*Aspergillus* (part of combination therapy)*:* 70 mg on first day, then 50 mg daily for ≥6 weeks. *Candida* [65]: 70 mg on first day, then 50 mg daily; duration dictated by extent of dissemination and resolution of signs/symptoms. *Mucorales* (part of combination therapy): 70 mg on first day, then 50 mg daily for a prolonged duration.
Anidulafungin	*Aspergillus* (alternative) [32]. *Candida* *Mucorales* (alternative in combination with amphotericin B) [66].	None.	Hypo/hypertension, hypokalemia, hypomagnesemia, and peripheral edema [74].	*Aspergillus* (part of combination therapy): 200 mg on first day, then 100 mg daily for ≥6 weeks. *Candida*: 200 mg on first day, then 100 mg daily; duration dictated by extent of dissemination and resolution of signs/symptoms [65]. *Mucorales* (part of combination therapy): 200 mg on first day, then 100 mg daily for a prolonged duration.
Micafungin	*Aspergillus* (alternative) [32]. *Candida* *Mucorales* (alternative in combination with amphotericin B) [66].	Sirolimus AUC may increase by 21% [75]. Cyclosporine: 1.7-fold increase in cyclosporine serum concentrations [76,77].	Phlebitis [75].	*Aspergillus* (part of combination therapy): 100–150 mg daily for ≥6 weeks. *Candida* [65]: 100 mg daily; duration dictated by extent of dissemination and resolution of signs/symptoms. *Mucorales* (part of combination therapy): 100–150 mg daily for a prolonged duration.
Amphotericin B deoxycholate	*Aspergillus* (alternative); *Candida* (alternative); *Cryptococcus* (alternative); *Coccidioides* (alternative) [43]. *Blastomyces* (alternative).	None.	Dose-dependent nephrotoxicity, infusion reactions, transaminitis, hypokalemia, hypomagnesemia, and hypocalcemia [78].	*Aspergillus* ^i^ [5]: 1–1.5 mg/kg/day for ≥6 weeks. *Candida* ^i^ [40]: 0.5–0.7 mg/kg/day; duration dictated by extent of dissemination and resolution of signs/symptoms. *Cryptococcus* ^i^ [56] (in combination with flucytosine or fluconazole)*:* 0.7–1 mg/kg/day for ≥2 weeks followed by step-down therapy. *Coccidioides* ^i^ [42]: 0.5–1 mg/kg/day until clinical improvement followed by step-down therapy. *Blastomyces* ^i^ [43]: 0.7–1 mg/kg/day for 1–2 weeks followed by step-down therapy.
Liposomal amphotericin B	*Aspergillus* (alternative); *Candida* (alternative); *Cryptococcus*; *Mucormycosis*; *Coccidioides* [42]. *Histoplasma* [42]. *Blastomyces*.		Dose-dependent nephrotoxicity (less common than with amphotericin deoxycholate), infusion reactions, transaminitis, hypokalemia, hypomagnesemia, and hypocalcemia [79].	*Aspergillus* ^j^ [13]: 3–5 mg/kg/day for ≥6 weeks. *Candida* ^j^ [40]: 3–5 mg/kg/day; duration dictated by extent of dissemination and resolution of signs/symptoms. *Cryptococcus* ^j^ [41] (in combination with flucytosine or fluconazole)*:* 3–4 mg/kg/day for ≥2 weeks followed by step-down therapy. *Mucorales* ^j^ [66]: 5–10 mg/kg/day for a prolonged duration. *Coccidioides* ^j^ [42]: 3–5 mg/kg/day until clinical improvement followed by step-down therapy. *Histoplasma* ^j^ [42]: 3–5 mg/kg/day for 1–2 weeks followed by step-down therapy. *Blastomyces* ^j^ [43]: 3–5 mg/kg/day for 1–2 weeks followed by step-down therapy.
Amphotericin B lipid complex	*Aspergillus* (alternative); *Candida* (alternative); *Cryptococcus*; *Mucorales*; *Coccidioides* [42]. *Histoplasma* [42]. *Blastomyces*.			*Aspergillus* ^k^ [12]: 5 mg/kg/day for ≥6 weeks [13]. *Candida* ^k^ [40]: 3–5 mg/kg/day; duration dictated by extent of dissemination and resolution of signs/symptoms. *Cryptococcus* ^k^ [56] (in combination with flucytosine or fluconazole)*:* 5 mg/kg/day for ≥2 weeks followed by step-down therapy [41]. *Mucorales* ^k^ [66]: 5–10 mg/kg/day for a prolonged duration. *Coccidioides* ^k^ [42]: 3–5 mg/kg/day until clinical improvement followed by step-down therapy. *Histoplasma* ^k^ [42]: 5 mg/kg/day for 1–2 weeks followed by step-down therapy. *Blastomyces* ^k^ [43]: 3–5 mg/kg/day for 1–2 weeks followed by step-down therapy.

Abbreviations: AUC: area under the curve; LTRs: lung transplant recipients; Cmin: minimum blood plasma concentration; Cmax: maximum blood plasma concentration; CNS: central nervous system. Dose adjustments indicated for estimated glomerular filtrate rate < 50 mL/min. (a) Weight-based dosing should be considered in obesity; (b) initial treatment for mild disease, otherwise amphotericin B lipid complex is indicated prior to initiation; (c) solution preferred; (d) goal trough level after 4–7 days of therapy (combined hydroxyitraconazole and itraconazole) of 0.5–3 mcg/mL; (e) goal trough level after 4–7 days of therapy of 1–5.5 mcg/mL; (f) use adjusted body weight for calculations; (g) consider combination therapy in severe disease; (h) goal trough level after ≥7 days of therapy of ≥1 mg/L; (i) use adjusted body weight for calculations but actual body weight can be considered for severe infections, suggested maximum dose of 150 mg daily; (j) use actual body weight for calculations; recommended maximum dose of 600 mg; (k) use actual body weight for calculations; recommend maximum dose of 500 mg.

**Figure 1:**
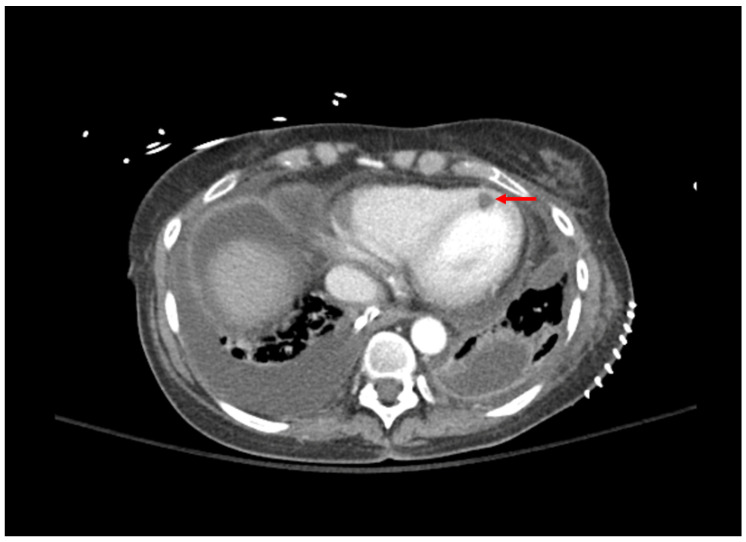
Computed tomography of the chest; bilateral pleural effusions grew *Aspergillus fumigatus*. Area of myocardial hypoperfusion consistent with fungal myocarditis is demonstrated by red arrow.

Bronchial anastomotic sites may undergo transient devascularization, and thus be more susceptible to ischemic injury. Thus, utilization of systemic agents may provide little benefit in patients with this complication in the setting of impaired blood flow to the ischemic site, and inhaled antifungal agents may be preferred. However, there is a low quality of evidence supporting the use of inhaled antifungals for IA [80]. Inhaled amphotericin B is often used as a prophylactic agent, but has demonstrated efficacy when used as an adjunct to systemic voriconazole, caspofungin, or amphotericin B for the treatment of IA [81]. Inhaled voriconazole has resulted in clinical improvement when utilized as monotherapy or adjunct to systemic caspofungin and liposomal amphotericin B [82,83]. It should be noted that the evidence is limited to case reports and case series for these agents, and further higher-quality studies are needed. A case vignette of a lung transplant recipient with aspergillosis is presented in Table 2 with the accompanying computed tomography image presented in Figure 1.

**Table 2:** Case vignettes of lung transplant recipients with Aspergillosis, Cryptococcosis, and Histoplasmosis.

Patient	Presentation	Diagnosis	Treatment	Outcome
1: *Aspergillosis*	53 YO, 5 weeks post-transplant. Received antithymocyte globulin and carfilzomib 2 weeks prior. Symptom of desaturations.	Chest CT: small right basilar empyema, partial collapse of left lower lobe, bilateral ground glass opacities, and septal thickening (Figure 1). Chest and pleural tissue culture from decortication procedure: *A. fumigatus*.	Voriconazole 6 mg/kg for 2 doses followed by 4 mg/kg daily. Voriconazole changed to liposomal amphotericin B after one week. Five weeks later, daily intrapleural voriconazole irrigation added for one week.	Systemic voriconazole stopped after one week for elevated hepatic function tests, intrapleural voriconazole stopped for bloody sputum. *Aspergillus* not redemonstrated in cultures. Patient death 4 months later due to bacterial sepsis.
2: *Cryptococcosis*	49 YO, 7 years post transplant. Received rituximab 3 months prior. Symptoms of headache, confusion, and photophobia.	MRI: hydrocephalus. CT chest: multifocal nodular abnormalities. LP: opening pressure 36 cm, CSF 65% neutrophils, protein 107, glucose 14, RBC 24, WBC 17. India ink stain: yeast. Serum and CSF antigen titer: ≥1:2560. CSF culture: *C. neoformans*.	Liposomal amphotericin 5 mg/kg + flucytosine for 16 days, then step-down to fluconazole 400 mg daily. Six days after step-down, liposomal amphotericin B restarted for altered mental status and cerebral swelling.	Progressive renal dysfunction to 1.5× baseline serum creatinine at time of fluconazole step-down. Death 48 h after cerebral swelling noted on imaging.
3: *Histoplasmosis*	38 YO, 14 years post transplant. Symptoms of low-grade fever and weight loss prompted colonoscopy.	CT chest/abdomen/pelvis: bowel wall thickening, lymphadenopathy, no acute pulmonary changes. Colonoscopy biopsy: *Histoplasma* (Figure 2). *Histoplasma* blood antibody: negative. *Histoplasma* urine antigen: 7.01 ng/mL.	Liposomal amphotericin 5 mg/kg for 7 days, then step-down to itraconazole 200 mg TID for 9 doses, followed by 200 mg BID Itraconazole trough 1.5 mcg/mL.	Remains on itraconazole after two years without significant side effects. Colonic thickening resolved five months after treatment initiation. *Histoplasma* urine antigen decreased to 0.77 ng/mL after 8 weeks of treatment.

Abbreviations: CSF: cerebrospinal fluid; CT: computed tomography; LP: lumbar puncture; MRI: magnetic resonance imaging; RBC: red blood cell count; TID: three times daily; WBC: white blood cell count; YO: years old.

**Figure 2:**
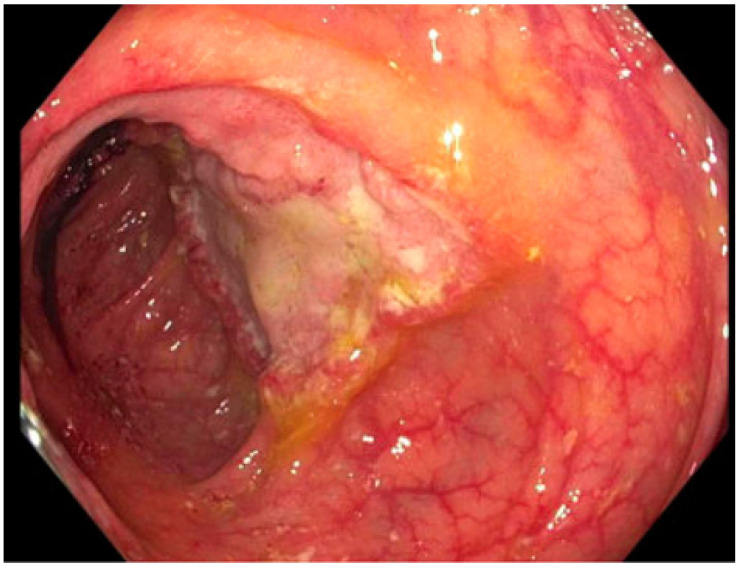
Ulcerated mass in the ascending colon; cold forceps biopsy yielded *Histoplasma capsulatum*.

## 3. *Candida*

*Candida* spp. often cause nosocomial infections in LTR. *Candida* infections commonly occur within the first 3 months post transplantation due to the presence of indwelling catheters, anastomotic dehiscence, and post-surgical complications [1]. The incidence of invasive candidiasis (IC) from 1980–2004 was 5.2%, and the most commonly isolated species were *Candida albicans*, *Candida glabrata*, and *Candida parapsilosis* [84]. Extracorporeal membrane oxygenation (ECMO) has been associated with a higher incidence of candidiasis, and risk factors for *Candida* infections include prolonged courses of antibiotic therapy, prolonged critical illness, and neutropenia. Because patients are often colonized with *Candida* spp., it can be difficult to identify the presence of a true *Candida* infection [85]. Therefore, isolated pulmonary manifestations of the disease are not typically identified, and IC can often manifest as candidemia, line infections, or surgical site infections.

### 3.1. Diagnosis

*Candida* pneumonia is rare and culture of *Candida* spp. from the airways of LTR usually reflect colonization but has been reported to be the cause of anastomotic infections in 2.8–9.8% of LTR in two case series [40,86,87]. Culture of *Candida* from a sterile site such as the blood provides a definitive diagnosis of candidiasis; however, the growth of *Candida* in culture is slow, with time to species identification typically being ≥50 h [88,89]. To circumvent the prolonged time to identification, several diagnostics have been developed.

Peptide nucleic acid fluorescent in situ hybridization assay (PNA-FISH) allows for rapid species identification of *Candida* in blood cultures in which different species fluoresce varying colors. The accuracy of PNA-FISH assays has been demonstrated to be ≥94% from blood cultures [90,91] and the use of PNA-FISH has also been reported for *Candida* identification from urine, catheter tips, and peritoneal fluid cultures [92]. 

PCR was compared to (1,3)-β-D-glucan assay in patients with IC which had similar diagnostic results except in deep-seated infections where PCR was more sensitive than (1,3)-β-D-glucan and both outperformed blood cultures [93]. A separate study evaluated the use of PCR compared to blood culture in critically ill patients demonstrating PCR sensitivity of 21.4% and specificity of 91.9% when compared to blood cultures, with the authors attributing a lower sensitivity compared to other studies to the use of serum instead of whole blood [94].

Matrix-assisted laser desorption ionization-time of flight mass spectrometry (MALDI-TOF MS) analysis detects proteins that are released by *Candida* spp. and compares those proteins to a database of yeast proteins. The use of MALDI-TOF MS on positive blood cultures also allows for rapid *Candida* species identification and has a reported sensitivity of 95.9% for *C. albicans* and 86.5% for non-albicans species [95,96].

T2Candida requires a specific diagnostic instrument (TDx) with magnetic resonance to detect *Candida* species in the blood and has a reported sensitivity of 89% in clinical practice [97].

(1,3)-β-D-glucan assays have also been studied in a non-SOT population; the sensitivity of Fungitell^®^ assay on serum samples was >80% for the diagnosis of *Candida* infections at a cutoff of 60 pg/mL, except for *C. parapsilosis*, whereby sensitivity was 72% [98]. A more recent article assessing (1,3)-β-D-glucan on candidemia revealed *C. auris* had a much lower sensitivity of 43.75% than other *Candida* spp. when ≥80 pg/mL was utilized as the cutoff for positivity [99]. Given the non-specific nature of (1,3)-β-D-glucan assays, it may be best used as a surveillance technique or in combination with standard blood cultures.

### 3.2. Treatment

Per IDSA guidelines, first-line agents for the treatment of candidemia are either fluconazole or echinocandins, depending on disease severity [65]. In mild manifestations of the disease, fluconazole may be initiated. However, in moderate–severe manifestations, it is recommended for a patient to receive an echinocandin until they are deemed clinically stable before transitioning to fluconazole. Certain *Candida* species, such as *C. glabrata*, are intrinsically resistant to fluconazole, and would thus be managed with an echinocandin, unless the patient is intolerant to echinocandins [100]. In a study that compared anidulafungin to fluconazole for the management of IC, 75.6% of patients in the anidulafungin group achieved treatment success vs. 60.2% in the fluconazole group (95% CI, 3.9 to 27.0). Patients were on immunosuppressive therapy in 14% of the anidulafungin group and 23% of patients in the fluconazole group [101]. Confirmation of superiority of echinocandins over fluconazole for IC was confirmed in a recent meta-analysis in non-SOT patients [102]. Additionally, a randomized, double-blind study found that micafungin was non-inferior to liposomal amphotericin B in achieving both clinical and mycological response, while also resulting in a decreased rate of adverse drug reactions, but SOT recipients only comprised 4–8% of the study population [103]. Therefore, further studies may be warranted to further solidify the role of echinocandins versus fluconazole in LTR with IC.

Alternative strategies may be considered in patients who cannot tolerate fluconazole or echinocandins. Liposomal amphotericin B may be utilized but carries an increased risk of significant adverse drug reactions. Voriconazole may be a possible option in patients with *C. krusei* or voriconazole-susceptible *C. glabrata* based on in vitro data [104]. However, there have been observations that voriconazole achieves subtherapeutic plasma concentrations in cystic fibrosis LTR, so therapeutic drug monitoring is recommended in this population [105]. Itraconazole has a similar spectrum of activity against *Candida* species compared to fluconazole but has a variable pharmacokinetic profile depending on the formulation used, leading to an unpredictable therapeutic effect [65]. Isavuconazole has been evaluated for IC treatment. In the ACTIVE trial, isavuconazole did not meet its non-inferiority endpoint when compared to caspofungin for IC treatment; however, the study excluded patients with severe immunodeficiencies [106]. Posaconazole and isavuconazole both exhibit an excellent spectrum of activity that is similar to fluconazole against most *Candida* species, but given the lack of evidence to support their use in LTR, fluconazole or echinocandins should be explored first prior to their use. Itraconazole has not been studied in IC and is currently not recommended for its management given fluconazole’s improved ease of administration, tolerability, and pharmacokinetic profile [65]. Table 1 contains information on antifungal interactions, adverse reactions, and recommended dosing for candidiasis.

## 4. *Cryptococcus*

Cryptococcal infections are estimated to occur in about 2.8% of SOT recipients [107]. The median time of occurrence is 16–21 months post-transplant, which corresponds to a longer time of onset compared to the previously discussed infections [108]. In a multicenter study of cryptococcal infections, 54% were identified as having pulmonary infections, 52.2% had CNS involvement, and 8.1% had skin/soft tissue/osteoarticular infections [107]. In a study of kidney transplant patients, presenting symptoms in cases of cryptococcal meningitis were headache, focal neurological signs, fever, and vomiting [109]. Cryptococcal pneumonia typically radiographically presents as solitary or multiple nodules. In SOT, infections are usually disseminated at the time of presentation [41].

### 4.1. Diagnosis

It is recommended to evaluate blood, urine, and cerebrospinal fluid (CSF) to determine the presence and extent of *Cryptococcus* infections which ultimately guides therapy [41]. Antigen assays using latex agglutination (LA) or lateral flow device (LFD) detect polysaccharides that are released by *Cryptococcus*. LA is more labor intensive and less sensitive for *C. gattii*, so LFD is the preferred method of antigen detection [41]; however, LFD can be impacted by a prozone effect in cases with a high disease burden [110]. In a study of SOT recipients with pulmonary cryptococcal infections, a positive serum antigen was present in 83.3% of cases; those with disseminated infections were more likely to have a positive serum antigen with higher antigen titers. A positive antigen was also more likely if there were ≥1 pulmonary nodules present [111]. A meta-analysis in the HIV population demonstrated a positive serum antigen was associated with *Cryptococcus* meningitis with a sensitivity of 99.7% and specificity of 94.1% [112]. Antigen presence in the CSF and serum is preferred for the diagnosis of *Cryptococcus* infections; lack of serum antigen does not necessarily exclude CNS involvement in SOT recipients [41].

PCR panels may also be useful for the identification of a wide range of organisms from the CSF including *C. neoformans* and *C. gattii* [113,114].

Isolation of *Cryptococcus* from culture, using Gomori’s methenamine silver or periodic acid-Schiff staining, may also be utilized for diagnosis [41].

### 4.2. Treatment

Management of cryptococcal infections is stratified depending on if the disease is localized to pulmonary tissue or if it has disseminated to the CNS. In patients with mild to moderate isolated pulmonary infections, fluconazole may present an appropriate therapeutic option. However, amphotericin B and flucytosine are recommended in CNS, disseminated, or moderate to severe pulmonary cryptococcal infections for at least two weeks followed by fluconazole step-down for consolidation and maintenance treatment [41]. A study comprising 299 HIV-positive patients with cryptococcal meningitis identified a statistically significant decrease in day 70 mortality in patients who received amphotericin B and flucytosine versus amphotericin B monotherapy [115]. An extended duration of treatment is indicated, with the 2010 IDSA guidelines suggesting a treatment duration of 6–12 months for SOT recipients [56]. Voriconazole, itraconazole, and posaconazole have demonstrated efficacy against *Cryptococcus* spp. in vitro, indicating that they may act as a suitable alternative to fluconazole in patients who cannot tolerate it [116,117]. *C. neoformans* carries an underlying resistance mechanism against echinocandins, rendering this pharmacological class ineffective [118]. Ultimately, further clinical trials are warranted to assess the efficacy of antifungal classes in LTR with cryptococcal infections. Table 1 contains information on antifungal interactions, adverse reactions, and recommended dosing for cryptococcosis.

Interestingly, the administration of calcineurin inhibitors (CNI), such as tacrolimus and cyclosporine, has been identified as improving survival in SOT recipients with cryptococcal infections. In a study of 111 SOT recipients with cryptococcosis, receipt of a calcineurin inhibitor was independently associated with lower mortality in multivariable analysis [107]. A case vignette of a lung transplant recipient with cryptococcosis is presented in Table 2.

## 5. Mucormycosis

The incidence of mucormycosis is highest within the first year post-lung transplant, with several cases developing in the first month [119]. In a study of 58 SOT recipients with mucormycosis, pulmonary manifestations developed in 53% of patients, and were associated with a 45.2% 90-day mortality after treatment [120]. A similar mortality rate of 57.1% for pulmonary mucormycosis was identified in a recent meta-analysis [121]. The high incidence of pulmonary involvement has significant ramifications for LTR. A systematic review of mucormycosis infections in LTR identified that 78% of mucormycosis developed in the first post-transplant year, and although patients received treatment with either posaconazole, amphotericin B, or a combination of the two medications, mortality was 32% [119]. The most commonly implicated sites of infection are the sinuses, lungs, and skin [122]. Tissue necrosis from hyphae invasion of vasculature causes most of the symptoms associated with mucormycosis. Within the SOT population, symptoms of pulmonary mucormycosis infection were most commonly fever in 54.8%, followed by cough in 29%, dyspnea in 19.4%, chest pain in 12.9%, and hemoptysis in 9.7%. In the same study, radiological findings were consolidation or mass lesions in 29%, nodules in 25.8%, cavitation in 22.6%, and infiltrates in 19.4% [120].

### 5.1. Diagnosis

Direct examination of specimens can be undertaken in the diagnosis of mucormycosis. The organism’s presence in tissue or fluids appears as irregularly branching non- or sparsely septate hyphae. Culture growth allows for speciation and antifungal susceptibility testing. Although DNA-based testing is being developed, there is no standardized assay currently available [66].

### 5.2. Treatment

Liposomal amphotericin B or amphotericin B lipid complex are recommended as first-line therapy in SOT patients in European guidelines [66]. Isavuconazole has demonstrated similar efficacy to amphotericin B in the VITAL study, although only 1 SOT patient out of 21 patients administered isavuconazole for primary treatment of mucormycosis was included [123]. A post hoc analysis of the VITAL study demonstrated day 84 survival of 63.6% in patients treated with isavuconazole for *Mucorales* CNS infection [124]. Posaconazole can also be considered a second-line agent in patients who fail or are unable to tolerate amphotericin B [125]. In patients with progressive disease despite amphotericin B treatment, isavuconazole or posaconazole may be considered for salvage therapy [66]. Table 1 contains information on antifungal interactions, adverse reactions, and recommended dosing for mucormycosis.

Deferasirox is an iron chelator that can be considered an add-on therapy for mucormycosis. This agent is typically utilized for patients who develop iron overload in transfusion-dependent anemia, but has been utilized in combination with other antifungal agents in patients who are refractory to liposomal amphotericin B [126]. Echinocandins have also demonstrated benefits when used in combination with amphotericin B [127,128]. Adjunctive inhaled amphotericin B has been employed in the treatment of pulmonary mucormycosis in stem cell transplant recipients and could be an area of future investigation in the LTR population [129].

## 6. Coccidiodiomycoses

Coccidiodomycosis is endemic to the southwestern United States, northern Mexico, and Central and South America. The causative organism is either *Coccidioides immitis* or *Coccidioides posadasii* [130]. Coccidioidomycosis occurs in 1.4–6.9% of SOT recipients in endemic regions [42]. Risk factors include allograft rejection, positive serology at the time of transplant, African American race, and a history of *Coccidioides* infection [42]. In general populations, most patients experience asymptomatic seroconversion; the incidence of asymptomatic seroconversion in SOT recipients is not known [131]. Proper identification of coccidioidomycosis infection is important in LTR as immunosuppressed patients are at an increased risk of disseminated disease. Symptoms of active *Coccidioides* infection in SOT recipients are usually fever or pneumonia but may also include the CNS, skin, or osteoarticular systems; pulmonary manifestations may include chills, night sweats, cough, dyspnea, and pleurisy [42].

### 6.1. Diagnosis

Radiographic findings of *Coccidioides* pneumonia that may be present include lobar consolidation, pulmonary nodules, mass-like lesions, interstitial infiltrates, or cavities [42]. *Coccidioides* spp. have a characteristic spherule with endospores. Positive cultures are definitive for diagnosis but may take 5–7 days for growth.

EIA testing for anti-coccidioidal IgM and IgG is widely available and is more sensitive than complement fixation and immunodiffusion; a positive IgM without positive IgG is not diagnostic and repeat testing over subsequent weeks is recommended [50]. The IgG titer may indicate a more severe disease and will turn negative once the infection is adequately treated [132]. Antigen presence in the serum or urine may be positive in cases of extensive infections [49].

### 6.2. Treatment

Per the 2019 AST Infectious Diseases Community of Practice guidelines, in patients with acute or chronic pulmonary coccidioidomycosis, fluconazole is recommended. If symptoms are severe or rapidly progressing, amphotericin B should be considered. In cases of meningeal *Coccidioides* infection, high-dose fluconazole is recommended [42]. Intrathecal amphotericin B has been utilized in patients with meningeal disease as well, and while it has achieved treatment success, it has also been associated with increased adverse drug effects compared to triazole antifungals [133,134]. In the non-SOT population, itraconazole has demonstrated similar efficacy compared to fluconazole in nonmeningeal coccidioidomycosis [135]. Alternative triazole antifungals such as voriconazole, isavuconazole, and posaconazole have been utilized, with voriconazole and posaconazole displaying efficacy in the management of refractory disease [57,136]. Echinocandins have only been reported for use in *Coccidioides* infection in a pediatric case series when used in combination with voriconazole [137,138]. Treatment duration is prolonged, with at least six months of treatment recommended followed by lifelong suppression, as relapses of infection have been reported [42]. Table 1 contains information on antifungal interactions, adverse reactions, and recommended dosing for *Coccidioides* infection.

## 7. Histoplasmosis

Histoplasmosis is endemic to the Midwestern United States, Mexico, and certain regions of South America and is thought to occur in less than 0.5% of SOT recipients in these endemic areas [1]. Symptoms of infection in SOT patients are typically non-specific and may be disproportionate to disease severity, but usually involve fever with imaging evidence of extra-pulmonary infection; infections indicative of progression include hepatosplenomegaly, pneumonia, GI disturbances, pancytopenia, weight loss, transaminase elevations, and lactate dehydrogenase elevations [42].

### 7.1. Diagnosis

Culture is the gold standard for diagnosis, but the growth of *Histoplasma* can take weeks and delay diagnosis. The histopathological presence of *H. capsulatum* cannot distinguish between active, past, and resolved infections. The histochemical stains Gomori methenamine silver and periodic acid-Schiff best highlight the cell wall and help differentiate *H. capsulatum* from other organisms; mucicarmine stains the capsule of *H. capsulatum* and differentiates from *Cryptococcus* and *P. jirovecii*, specifically [139].

The use of *Histoplasma* antigen assay of BAL fluid in mainly non-transplant recipients was evaluated and demonstrated a positive result in 93.5% of patients with histoplasmosis; when combined with BAL cytopathology, there was a sensitivity of 96.8% for diagnosis of histoplasmosis [140]. Antigen detection in the urine is marginally more sensitive than in the serum; combining the two tests increased the ability to detect *Histoplasma* antigen to 82.8% [141] and is recommended by the AST Infectious Diseases Community of Practice guidelines [42]. However, a more recent study with paired urine and serum antigen testing identified a 98% agreement, making the argument that a single test may be appropriate for initial histoplasmosis screening [142]. Isolated pulmonary histoplasmosis demonstrates lower antigen sensitivity than disseminated histoplasmosis. Antigen levels decline with treatment and thus can be utilized as a marker of treatment success. It should be noted that *Histoplasma* antigen has cross-reactivity with *Blastomyces dermatitidis*, *Paracoccidioides brasiliensis*, and *T. marneffei* [139].

Antibodies to *Histoplasma* can be detected after several weeks via immunodiffusion, complement fixation, and EIA testing but sensitivity in the SOT population is low and of limited utility [42,143]. Complement fixation is typically considered presumptively positive if the titer is ≥1:8 but this could indicate a past infection; to diagnose an acute infection, a 4-fold increase in the titer (taken 2 weeks apart) or a titer of ≥1:32 is required [144,145].

### 7.2. Treatment

Therapies are highly effective if they are initiated before the infection becomes severe. Mild to moderate infections may be treated with itraconazole monotherapy for an extended duration of at least 12 months. For severe infections, liposomal amphotericin B should be employed followed by itraconazole. In a study of 152 SOT recipients, of which 5% were LTR, amphotericin B followed by step-down to triazoles was utilized in 73% of patients which resulted in 90% survival. The different triazoles that were utilized as step-down therapy included itraconazole, voriconazole, or fluconazole, although fluconazole was only utilized in one of the patients [146]. In a survey of infectious disease physicians, step-down therapy for severe *Histoplasma* infections (non-CNS) was reported to be itraconazole, voriconazole, posaconazole, isavuconazole, or fluconazole [147]. For refractory patients or those who do not tolerate first-line agents, posaconazole, voriconazole, and isavuconazole are recommended as salvage therapy over fluconazole [42]. Lifelong antifungal prophylaxis may not be necessary if the patient maintains adequate allograft function and is maintained on low-dose immunosuppression. Consideration of lifelong suppression with itraconazole should be weighed against its adverse drug effect profile, which includes possible heart failure exacerbations and hepatitis as well as its drug–drug interactions with CNIs. *H. capsulatum* is resistant to echinocandins and may develop resistance to fluconazole and voriconazole [148,149]. Table 1 contains information on antifungal interactions, adverse reactions, and recommended dosing for *Histoplasma* infections.

When utilizing itraconazole, an appropriate understanding of the pharmacokinetic variability among different dosage forms is required. There are currently two dosage forms available: oral capsules and oral suspensions; these dosage forms are not interchangeable. Itraconazole capsules require a low gastric pH for dissolution and should thus be taken on an empty stomach to improve absorption. This also results in increased variability in bioavailability, which may lead to unpredictable treatment effects across patients. However, the suspension, while absorbed better, is associated with increased GI adverse effects [32]. A newer capsule formulation, super bioavailable (SUBA) itraconazole, has improved absorption compared to traditional capsule formulations as well as reduced variability in bioavailability [150]. SUBA-itraconazole continues to have diminished bioavailability if taken with meals, so it is recommended to be taken in a fasted state. A case vignette of a lung transplant recipient with histoplasmosis is presented in Table 2, with a biopsy image presented in Figure 2.

## 8. Blastomycosis

*Blastomyces dermatitidis* is the causative organism for blastomycosis and it exists in states that border the Mississippi River basin, Great Lakes, St. Lawrence Seaway, as well as in Ontario and Manitoba [42]. Complement inhibitors, such as eculizumab, have been reported to increase the risk of blastomycosis in LTR [151]. The most common presentation in patients with blastomycosis is pneumonia and this progresses in some instances to acute respiratory distress syndrome. The respiratory tract is involved in the majority of *Bloastomyces* infections in SOT recipients, typically presenting as fever and cough; radiographically, patients may have lobar or interstitial infiltrates with mediastinal adenopathy or lung cavitations. Outside of the lungs, organs that can be involved in disseminated infections include the skin, genitourinary tract, osteoarticular systems, and, rarely, the CNS.

### 8.1. Diagnosis

Culture is the most definitive way to diagnosis blastomycosis but requires 4–6 weeks of incubation. Histopathological identification of *Blastomyces* can occur via visualization of micro-abscesses and noncaseating granulomas; Gomori methenamine silver and periodic acid-Schiff stains can aid in the visualization of yeast forms. In respiratory specimens, KOH ± calcofluor white wet preparation can be useful for detection [152].

A chemiluminescent DNA test can identify *B. dermatitidis* within hours but is cross-reactive with *Paracoccidioides basiliensis*.

Antibody detection utilizing complement fixation has low sensitivity and specificity; immunodiffusion techniques are more sensitive. Antigen testing can be performed on urine, BAL, CSF, and serum but has a high cross-reactivity with *Histoplasma*, *Paracoccidioides*, and *T. marneffei* [153]. Serial antigen monitoring has been shown to correlate with treatment response [42].

### 8.2. Treatment

The 2008 clinical practice guidelines for the treatment of blastomycosis recommend that all immunocompromised patients receive treatment for blastomycosis [43]. Liposomal amphotericin B is recommended as a first-line therapy for severe pulmonary, CNS, or disseminated infections, and once stable the patient may step down to itraconazole for 6–12 months [43]. For CNS disease, voriconazole is preferred to itraconazole for step-down therapy due to better CNS penetration [42]. Itraconazole may be used as first-line therapy if the patient presents with mild or localized disease. Voriconazole and posaconazole have demonstrated favorable spectrum of activities against *B. dermatitidis* in vitro and thus can serve as alternatives to itraconazole [42,68]. Isavuconazole’s use in the treatment of blastomycosis has been described in two small case series [72,154]. Though the echinocandins seem to display some in vitro activity, this does not correspond to clinical efficacy and thus they do not have a current role in therapy [43]. Table 1 contains information on antifungal interactions, adverse reactions, and recommended dosing for *Blastomyces* infections.

## 9. Scedosporium/Lomentospora and Fusarium

*Scedosporium* and *Fusarium* are filamentous fungi that are associated with a high risk of mortality in immunocompromised patients [155,156]. Clinical manifestations range from keratitis and subcutaneous nodules to invasive infections such as brain abscesses and disseminated diseased [6,157]. In LTR, CT findings may include nodular ground glass infiltrates, bronchiectasis with tree-in-bud or ground glass nodules, or cavitation [158,159]. Possible risk factors for severe fusariosis include prolonged neutropenia and T-cell immunodeficiency [157]. The incidence of invasive disease has been reported to be 3–14% [158]. Given the high mortality rate reported in the hematological malignancy population [155,160], proper management of these infections is imperative in LTR.

### 9.1. Diagnosis

Culture isolation of *Fusarium* and *Scedosporium/Lomentospora* from tissue or bodily fluid yields a definitive diagnosis; histopathologically, *Fusarium* is similar to other hyalohyphomycetes [161]. *Fusarium* is more likely to grow in blood cultures when compared to *Aspergillus* spp. [157]. In *Furasium* infections, (1,3)-β-D-glucan assays may be positive but are non-specific; decreasing values could be useful for monitoring response to treatment [162]. *Aspergillus* galactomannan assay may also be positive in fusariosis [163].

### 9.2. Treatment

Management of these infections is complicated by the resistance profiles of some species, such as *Lomentospora prolificans* (previously known as *Scedosporium prolificans*), which are resistant to all available antifungals [164,165]. A single-center study evaluated *Scedosporium apiospermum* and *Lomentospora prolificans* in 30 LTR; posaconazole was their most commonly utilized agent for the management of infection [158]. Treatment of infection resulted in improved lung function over 6 months, with a median duration of therapy of 364 days. A study in non-transplanted cystic fibrosis patients demonstrated that combination therapy with an echinocandin plus voriconazole/posaconazole was superior to monotherapy in *Scedosporium apiospermum* infections [166]. Itraconazole has demonstrated in vitro activity against this pathogen, which lends itself as a possible alternative to other triazoles. Echinocandins and isavuconazole exhibit limited activity against *Scedosporium* and are thus not recommended [167]. Interestingly, a case report identified that nebulized voriconazole is a possible option for the management of *Scedosporium apiospermum*, which can aid in limiting its adverse drug effect and drug interaction profile due to a lack of systemic absorption. Further studies are warranted to fully assess the efficacy and safety of this therapeutic option, however.

*Fusarium* may similarly be treated with broader-spectrum triazoles such as voriconazole and posaconazole [157]. Lipid formulations of amphotericin B have also been utilized, but awareness of the adverse drug effect profile should be maintained when considering it over triazoles. When applicable, surgical debridement should also be considered, as should secondary prophylaxis [161]. A case series of 6 LTR with *Fusarium* infections recommended either a combination of amphotericin B and voriconazole or amphotericin B monotherapy as first-line; posaconazole can be used in a refractory disease [168]. *Fusarium* spp. have intrinsic resistance against echinocandins, limiting the use of these agents. Itraconazole may act as a possible alternative, but evidence in LTR is limited. Isavuconazole’s in vitro activity against *Fusarium* revealed MIC’s >16 ug/mL, which limits its applicability against this group of pathogens [169]. There is currently limited evidence to support the use of fluconazole as well. Despite the current evidence supporting these recommendations, certain multidrug-resistant strains of *Fusarium solani* have also been shown to be resistant to voriconazole, caspofungin, and posaconazole in vitro which complicates drug selection [164]. Table 1 contains information on antifungal interactions, adverse reactions, and recommended dosing for infections due to *Scedosporium/Lomentospora* and *Fusarium* infections.

## 10. PJP

*Pneumocystis jirovecii* pneumonia (PJP) may develop in both the general and immunosuppressed population. The incidence of PJP varies from 3–15% depending on the type of transplant as well as the use of prophylaxis, with lung transplantation having the highest incidence of PJP among SOT recipients [170]. A key risk factor for PJP is the receipt of corticosteroids, T-cell depletion, neutropenia, and the presence of CMV infections. The highest incidence of PJP occurs in the first 1–6 months post-transplant, often warranting prophylaxis within that time period [170]. However, reports of infection after 12 months post-transplant have occurred. Asymptomatic isolation of *P. jirovecii* in LTR may exceed 10%. Symptoms typically involve progressive dyspnea, fever, and cough [170].

### 10.1. Diagnosis

Chest radiographs are non-diagnostic for PJP, presenting as diffuse interstitial processes [170]. Isolation of PJP from the respiratory tract can provide a definitive diagnosis; BAL is more sensitive than sputum. Silver, polychrome, or Calcofluor white stains provide the ability to exclude PJP from BAL samples. [170] Gomori methenamine silver stain has been reported to have a lower diagnostic yield in non-HIV immunocompromised patients when compared to the HIV population [171]. The classic histopathological finding of PJP includes foamy eosinophilic exudate with a honeycomb appearance; patchy distribution throughout the lungs is common [170].

Direct immunofluorescent antibody (DFA) staining on sputum or BAL samples is the most reliable method for the identification of PJP and should be employed for initial diagnoses [170].

PCR on BAL samples has demonstrated high agreement with DFA [172,173,174]; in non-HIV patients, a PCR cycle threshold value of <31 excluded colonization and value >35 excluded PJP altogether, although the cutoff for positive cycle threshold differs by assay [173,174,175].

(1,3)-β-D-glucan assay on serum samples has been reported to have a sensitivity of 86% but with a specificity of only 83% in non-HIV patients for the diagnosis of PJP [176].

### 10.2. Treatment

First-line therapy for PJP is trimethoprim–sulfamethoxazole. Careful monitoring of possible adverse drug reactions such as hyperkalemia, neutropenia, and thrombocytopenia should be observed when utilizing this agent. Alternative strategies such as primaquine and clindamycin, pentamidine, atovaquone, and dapsone are not as effective as trimethoprim–sulfamethoxazole in the treatment of PJP [170]. These agents are often considered in situations in which a patient is not a candidate for trimethoprim–sulfamethoxazole, such as in those with sulfa allergies. Primaquine and clindamycin monotherapy is typically reserved for a milder manifestation of disease. Prior to the use of primaquine or dapsone, patients must be screened for G6PD deficiencies due to the risk of hemolytic anemia. Atovaquone can be used in mild to moderate disease manifestations, but can be associated with dermatologic adverse drug reactions such as skin rashes.

Pentamidine may be utilized as either an IV or nebulized formulation, with nebulized being reserved for prevention as it is unlikely to reach adequate concentrations in the distal airways needed for the treatment of PJP. Pentamidine is associated with significant side effects including hypo- and hyper-glycemia, neutropenia, thrombocytopenia, GI side effects, as well as pancreatic islet cell necrosis.

Echinocandins are possible options in cystic manifestations of PJP. Combination therapy of echinocandins with trimethoprim–sulfamethoxazole may be considered due to their possible synergy in severe PJP, but the overall quality of evidence and level of recommendation by the American Society of Transplantation’s guidelines is weak [170].

Adjunctive corticosteroids should be considered early in the treatment course in cases of hypoxia, defined as arterial oxygen partial pressure of >70 mmgHg or alveolar-arterial gradient of <35 mmHg on room air [170,177]. Table 1 contains information on antifungal interactions, adverse reactions, and recommended dosing for PJP infections.

## 11. Inhaled Antifungals

Inhaled antifungal therapy is an attractive option for the treatment of isolated pulmonary fungal infections due to low systemic absorption which minimizes drug interactions and adverse effects. Nebulized amphotericin B or liposomal amphotericin B have been reported as adjunctive therapies for the treatment of tracheobronchial aspergillosis, invasive pulmonary aspergillosis, as well as pulmonary infections due to *Mucorales* spp., *Scedosporium* spp., and *Fusarium* spp., and is recommended as an adjunctive therapy in cases of tracheobronchial aspergillosis that are associated with ischemic airways in lung transplant recipients by the IDSA and AST-IDCOP [32,178]. Inhaled voriconazole has been reported in case series of *Aspergillus* infections, either as monotherapy or adjunctive to systemic antifungal therapy, resulting in good clinical response [82,83]. The feasibility of nebulizing posaconazole has also been reported, including a case report of using inhaled posaconazole as an adjunct during bronchoscopies in three lung transplant patients with *Scedosporium apiospermum* [83,179]. Inhaled opelconazole has been studied for the treatment of fungal infections caused by *Aspergillus* spp., *Candida* spp., and *Rhizopus* in pre-clinical murine models and is currently under investigation for the treatment of invasive aspergillosis in lung transplant recipients [180].

## 12. Conclusions

Fungal infections following lung transplant continue to be a source of significant morbidity, with LTR being at particularly high risk for fungal infections among solid organ transplant recipients due to the potential for ischemic airway injury and communication of the allograft with the environment. It is also challenging to differentiate between colonization and active infection when species are identified within the lung allograft. Prompt diagnosis and treatment are integral to limiting allograft damage. Amphotericin B continues to be a mainstay of treatment for severe fungal infections, although newer triazole antifungals may be appropriate in some cases and carry a lower risk for side effects. There are many drug interactions between immunosuppressives and triazole antifungals as well as additive adverse effects that must be taken into consideration when selecting treatment options for fungal infections in LTR. Inhaled antifungals are promising due to their direct application to the site of infection which may diminish side effects and drug interactions, although further studies in larger lung transplant populations are warranted.

## Data Availability

Not applicable given article type (review).
